# Winter Burst of Pristine Kashmir Valley Air

**DOI:** 10.1038/s41598-018-20601-z

**Published:** 2018-02-20

**Authors:** Zainab Q. Hakim, Gufran Beig, Srinivas Reka, Shakil A. Romshoo, Irfan Rashid

**Affiliations:** 1 Indian institute of Tropical Meteorology, Pune, India; 2 Department of Earth Science, University of Kashmir, Srinagar, India; 3 Present Address: Centre for Atmospheric Sciences, University of Cambridge, Cambridge, UK

## Abstract

The Kashmir Valley in India is one of the world’s major tourist attractions and perceived as a pristine environment. Long term monitoring of fine particulate matter, PM_2.5_ (particles having aerodynamic diameter of 2.5 μm or less), responsible for deteriorating human health, has been done for the period 2013–14. Results indicate that air quality of the capital city Srinagar (34.1°N, 74.8°E) deteriorates significantly in particular during winter, where level of PM_2.5_ touches a peak value of 348 μg/m³ against the Indian permissible limit of 60 μg/m³. The emissions due to domestic coal usage are found to be 1246.4 tons/yr, which accounts for 84% of the total annual emissions. The on-line high-resolution weather research and forecasting model with embedded chemistry module (WRF-Chem), which accounts for emission inventory developed in this region reproduced the seasonal variability reasonably well. Cold temperatures with dry conditions along with elevated level of biofuel emissions from domestic sector are found to be the major processes responsible for winter period particulate pollution. The back trajectories show that westerly winds originating from Afghanistan and surrounding areas also contribute to the high PM_2.5_ levels.

## Introduction

The high altitude destinations around the world are perceived to have a clean environment. They become preferred places for tourist attractions. However, such places are slowly found to be environmentally degrading due to ever increasing tourists and associated emissions. The geographical location of the region also plays an important role, which may result in long-range transport of pollutants. Owing to the meteorological conditions during winter, violation of National Ambient Air Quality Standards for PM_2.5_ has been observed on a consistent basis in pristine places such as the Salt Lake Valley in Utah^1^. One such place in California is the San Joaquin Valley, it experiences high levels on PM_2.5_ mass concentration and secondary aerosol compounds constitute over half of the PM_2.5_ mass in winter. Secondary aerosol formation in this region is significant during winter^2^.

The Srinagar region of Kashmir Valley in India with altitude of 1592 meters is a popular tourist destination for domestic as well as foreign tourists due to its salubrious environment. In recent times, Kashmir Valley has become the largest urban center across the whole Himalayan region and is undergoing areal expansion and facing high rates of population growth^3,4^. The increased urbanization and the increased use of biofuels in the valley are disturbing the environment. Studies show that lower mixing heights, limited dispersion and long-range transport of pollutants results in higher pollution levels during winter, as the pollutants get trapped in the lower layers of the atmosphere^5,6^. However, no systematic study has been done for this region, to understand the air quality and different factors responsible for its deterioration. This article makes an attempt to present the status of air quality in the capital city of Kashmir Valley, identifies the extreme air pollution events and uses the recently developed emission inventory along with WRF-Chem simulations to understand the processes responsible for this seasonal variability.

## Observed data and model details

**Figure 1:**
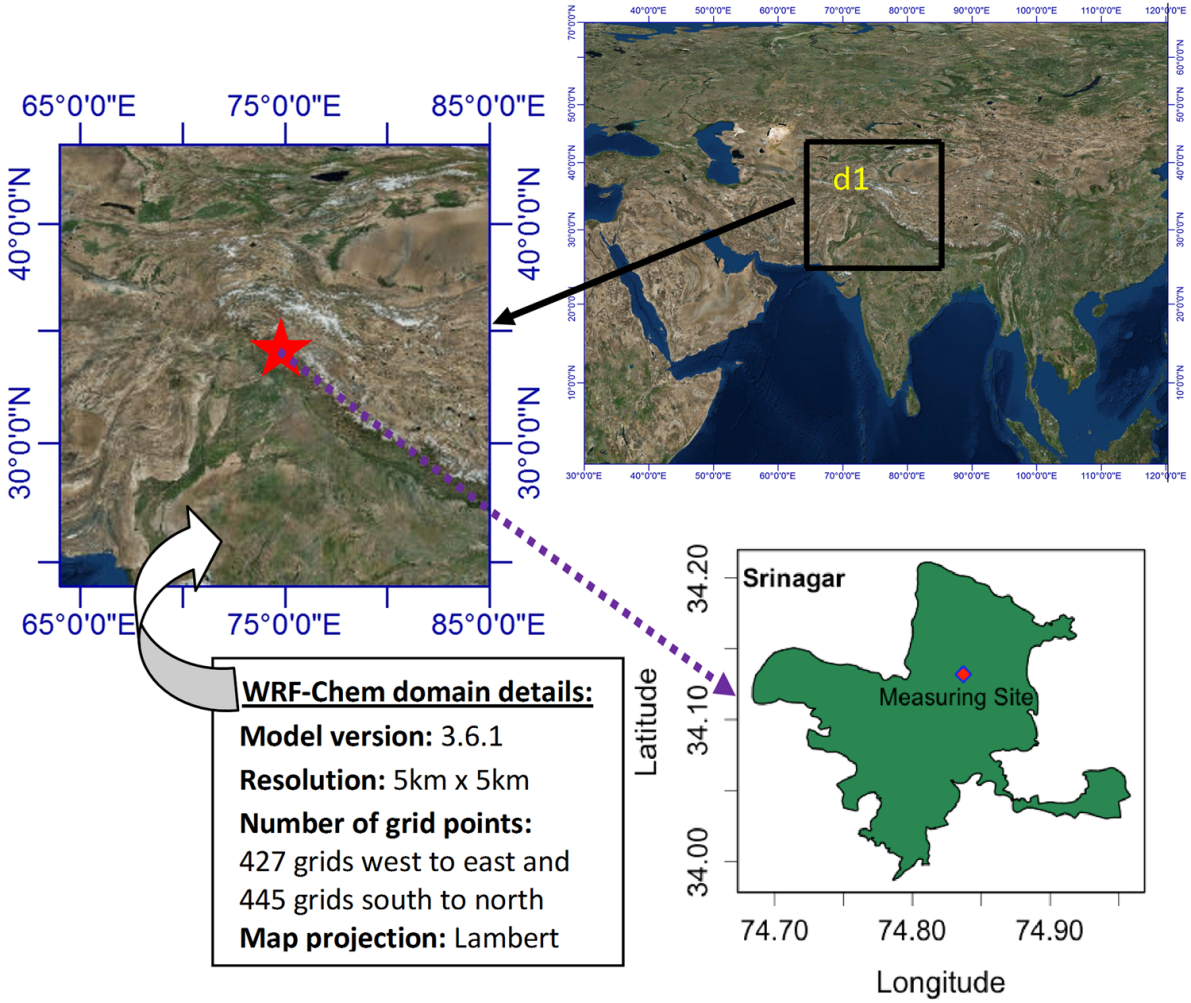
The WRF-Chem model (version 3.6.1) domain at 5 km horizontal resolution with 427 × 445 grid points indicated by the black solid rectangular box identified as d1. The location map of Srinagar and the measuring site developed using QGIS version 2.14.10-Essen [QGIS Development Team. Open Source Geospatial Foundation Project. http://qgis.osgeo.org] and the terrain showing map prepared using licensed ArcGIS version 9.3.1 software by Environmental Systems Research Institute (ESRI, www.esri.com).

In this work we have analyzed hourly observational data of PM_2.5_ collected for one year period (15 May 2013 to 20 April 2014). The data was obtained as part of the project Modeling Atmospheric Pollution And Networking (MAPAN) program undertaken by Indian Institute of Tropical Meteorology (IITM), Pune in collaboration with the University of Kashmir. Continuous Ambient Air Quality Monitoring Station (CAAQMS) has been installed in the Department of Earth Sciences of University of Kashmir. PM_2.5_ concentrations were measured using APM 550 MFC system, which uses WINS impactor for particle size separation and is based on impact or designs standardized by US-EPA for ambient air quality monitoring whose technical details can be found elsewhere (http://www.envirotechindia.com/apm-550-mfc.html). Figure 1 shows the details of WRF-Chem model domain, geographical location of Srinagar and the measuring site.

The contribution to PM_2.5_ levels by surrounding regions was investigated by tracing the path of the air parcels in different months over Srinagar (34.1°N, 74.8°E). The back trajectory analysis was performed with the Global Data Assimilation (GDAS) dataset at an altitude of 500 m above ground level (AGL) during the study period. The 500 m AGL height is commonly preferred for such trajectory analysis^7^ as it is considered suitable for the identification of medium/long-range transport impacts and the trajectory falls well within the atmospheric boundary layer^8–10^. The monthly average backward trajectories (a sequence of latitude and longitude values for the endpoints) in time (starting (ending) time 2300 (0000) UTC) were computed using version 4 of the HYSPLIT model^11,12^ (HYbrid Single-Particle Lagrangian Integrated Trajectory model) of NOAA’s-ARL (National Oceanic and Atmospheric Administration’s Air Resources Laboratory). The calculated back trajectories of air parcels were then used to better understand the pathways of pollutants from the medium/long-range distances in order to identify the source regions during the months in which the transport of pollutants was suspected. The back trajectory maps were created using ArcGIS version 9.3.1 software by Environmental Systems Research Institute (ESRI, www.esri.com) based on endpoints.

The Weather Research and Forecasting model coupled with Chemistry (WRF-Chem) has been set up as part of the System of Air quality and weather Forecasting And Research (SAFAR) project^13,14^ to conduct research on important pollutants in Srinagar. The model was driven by National Centers for Environmental Prediction Final (GFS/FNL) meteorological reanalysis fields as initial and lateral boundary conditions. Chemical boundary conditions were taken from Monitoring Atmospheric Composition and Climate (MACC) project of the European Centre for Medium-Range Weather Forecasts (ECMWF). Details of the preparation of input fields including emission inventories of SAFAR-India can be found elsewhere (http://eccad.sedoo.fr/). Important settings used in the configuration of WRF-Chem model for microphysics, longwave radiation, shortwave radiations, biogenic emissions and boundary conditions are described in previous studies^15^. The anthropogenic emissions are used from the SAFAR-India inventory at 0.5° × 0.5° resolution. The WRF-Chem model of version 3.6.1 was set-up to perform air quality research with the single domain (64°E to 85°E and 24°N to 44°N) covering Kashmir at a horizontal resolution of 5 km with 427 × 445 grid points and keeping Srinagar as center (Fig. 1). The model is configured in the Lambert conformal map projection. For the domain 27 vertical levels were used with a maximum height of 18 km.

The relative impacts of local emissions and long-range transport on the levels of PM_2.5_ have been studied by analyzing 3 different scenarios of the WRF-Chem interactive model simulations namely Scenario A, Scenario B and Scenario C, respectively. In Scenario A, the model was run with normal emissions and dynamics. Hence, Scenario A represents both local emissions and medium/long-range transport, which means that it includes medium/long-range transport of pollutants from surrounding regions. In Scenario B, local emissions i.e. anthropogenic and natural emissions, were switched off and made zero. Scenario C was computed by taking the difference between the results of Scenario A and Scenario B. Scenario C, thus, represents the impact of only local emissions on the levels of PM_2.5_ without accounting for any kind of transport. Similar analysis has been done over Delhi region. Results show that CO budget is influenced by long-range transport^14^.

## Methodology for Emission Inventory

**Figure 2:**
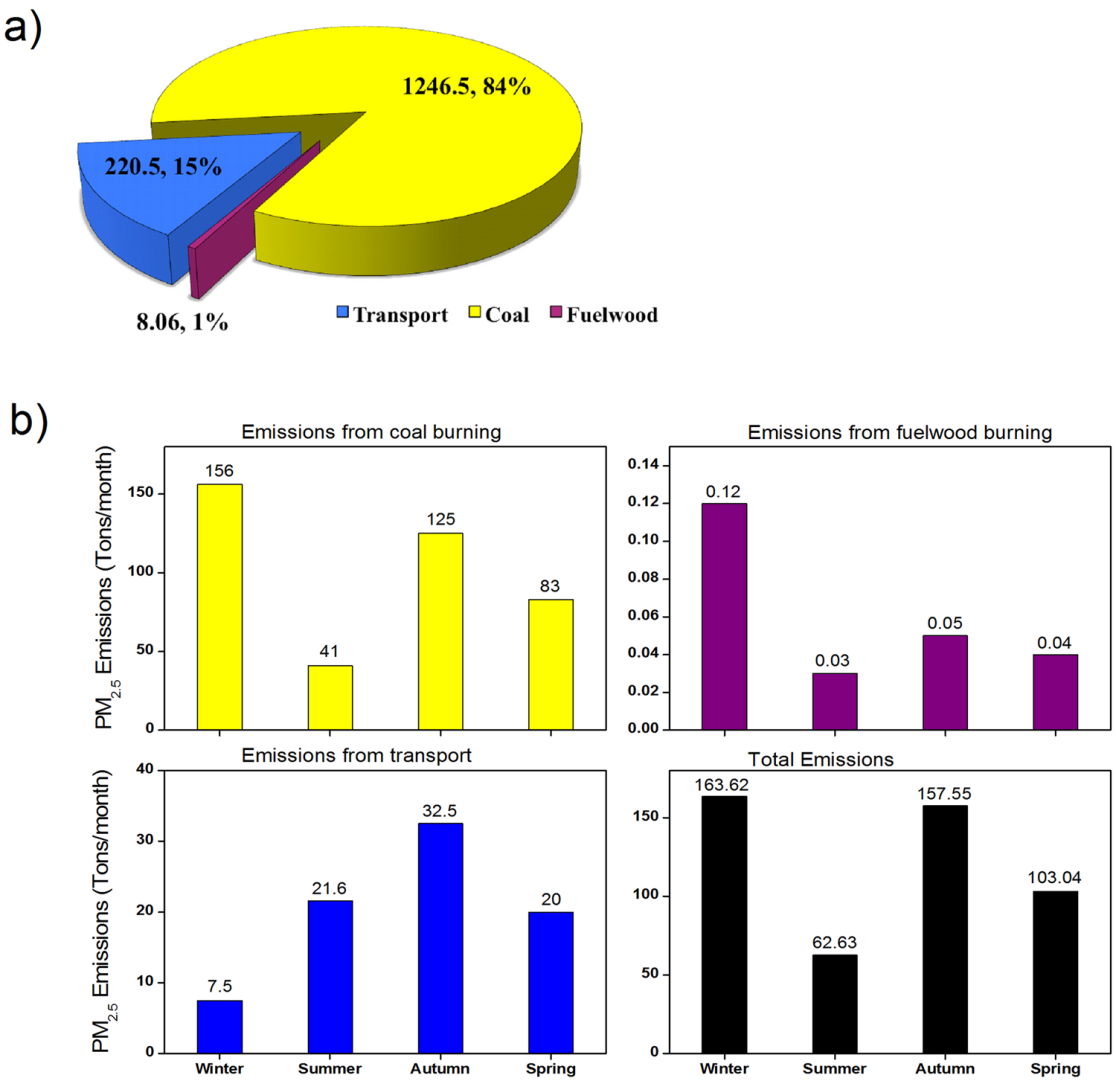
(**a**) Annual estimation of emissions of PM_2.5_ in tons per year and percentage share from different sources over Srinagar City. (**b**) Seasonal distribution of emissions of PM_2.5_ from coal, fuelwood burning, transport and total emissions from the three sources.

Emission inventory for Srinagar was developed for the year 2013–2014. We identified biofuel burning and transport sector to be the major sources of emission of PM_2.5_ in this region (Fig. 2a). The emissions from coal burning, fuel wood burning and vehicular combustion have been estimated in this study. The methodology is proven and details are provided and discussed earlier^16^. Emission of PM_2.5_ from a particular source has been estimated as a product of activity data and emission factors (EFs). The information about coal usage is classified data and has been procured from excise and taxation department of state government (http://jkexcise.nic.in/) under special provision. A total of 170285 tons of coal was imported in the Srinagar and Budgam district during the period of study. Together these regions comprise of a population of nearly 2,000,000 persons (http://www.census2011.co.in/census/district). Based on the assumption of uniform consumption of resources across the two regions, Srinagar, which comprises of nearly 60% of this population is expected to use 60% of the total coal imported in the two districts. The information about consumption of fuel wood has been collected during a field study as part of the emission inventory development. The usage of fuel wood has been estimated on the basis of the number of bakery shops and hamams in Srinagar city. Further, the use of fuel wood in the offices during winter has also been calculated based on the estimates of coal usage. Around 5253.4 and 123.4 tons of fuel wood were used in bakeries and hamams, respectively. The emission factors (EFs) for coal and fuel wood for fine particulate matter (PM_2.5_) used in the present study are 12.2 g/kg and 1.5 g/kg, respectively^17,18^. The count of different types of vehicles in the valley was also obtained as part of the field work from Regional Transport Office (RTO), Srinagar. For transport sector the EFs for PM of various types of vehicles, developed by the Air Quality Monitoring Project– Indian Clean Air Programme (ICAP) (Source Apportionment Studies, CPCB 2010, http://www.cpcb.nic.in/Emission_Factors) have been used for in the present work. These EFs are listed in Table 1. From local field study, it has been observed that the number of vehicles on road drops by almost half during winter as compared to summer and autumn, which are quite present and people venture out in full strength. The spring season normally remains pleasant in Srinagar but the temperature starts to fall during the end period and in road emissions start to decline little bit. Considering the above statistics it has been assumed that during winter, summer, autumn and spring the vehicular emissions contributes to about 15%, 30%, 30% and 25% respectively to the total annual emissions of PM_2.5_.

**Table 1:** Total number of vehicles and types of fuel used in them with the corresponding EFs for PM.

Sr. No.	Category	Fuel Type	Total	EFs for PM (g/hr)
1	2-Wheeler	Petrol	93087	0.013
2	3-Wheeler	Petrol	10365	0.015
		L/C	6009	0.015
	Total 3-Wheeler		16374	
3	Passenger Vehicles	Petrol	77340	0.015
	Passenger Vehicles	Diesel	77340	0.24
	Total Passenger Vehicles		154680	
4	Buses	Diesel	7012	0.24
5	Goods Vehicle			
	Trucks/HCV	Diesel	14635	0.42
	MLV/LGV	Diesel	4007	0.475
	Others	Diesel	842	0.42
	Others	Petrol	842	0.24
	Total goods vehicle		20326	

Figure 2a shows the annual emission estimation. It reveals that the emissions from coal burning are the highest that is around 1246.5 tons/year followed by the emissions from vehicular combustion that is 220.5 tons/year and the least are the emissions from fuel wood burning that is around 8.06 tons/year. Figure 2b depicts the approximate estimates of PM_2.5_ emissions (tons per month) from coal burning, fuel wood burning, vehicles and the total emissions from the three sources in different seasons. There is a significant difference in the emissions in different seasons. Usage of coal and fuel wood are the highest in winter, hence the emissions from these sources are also high during winter compared to other seasons. Total emissions of PM_2.5_ during winter (~163.62 tons/month) are the highest followed by the autumn (~157.55 tons/month), spring (~103.04 tons/month) and summer (~62.63 tons/month).

## Results and Discussions

**Figure 3:**
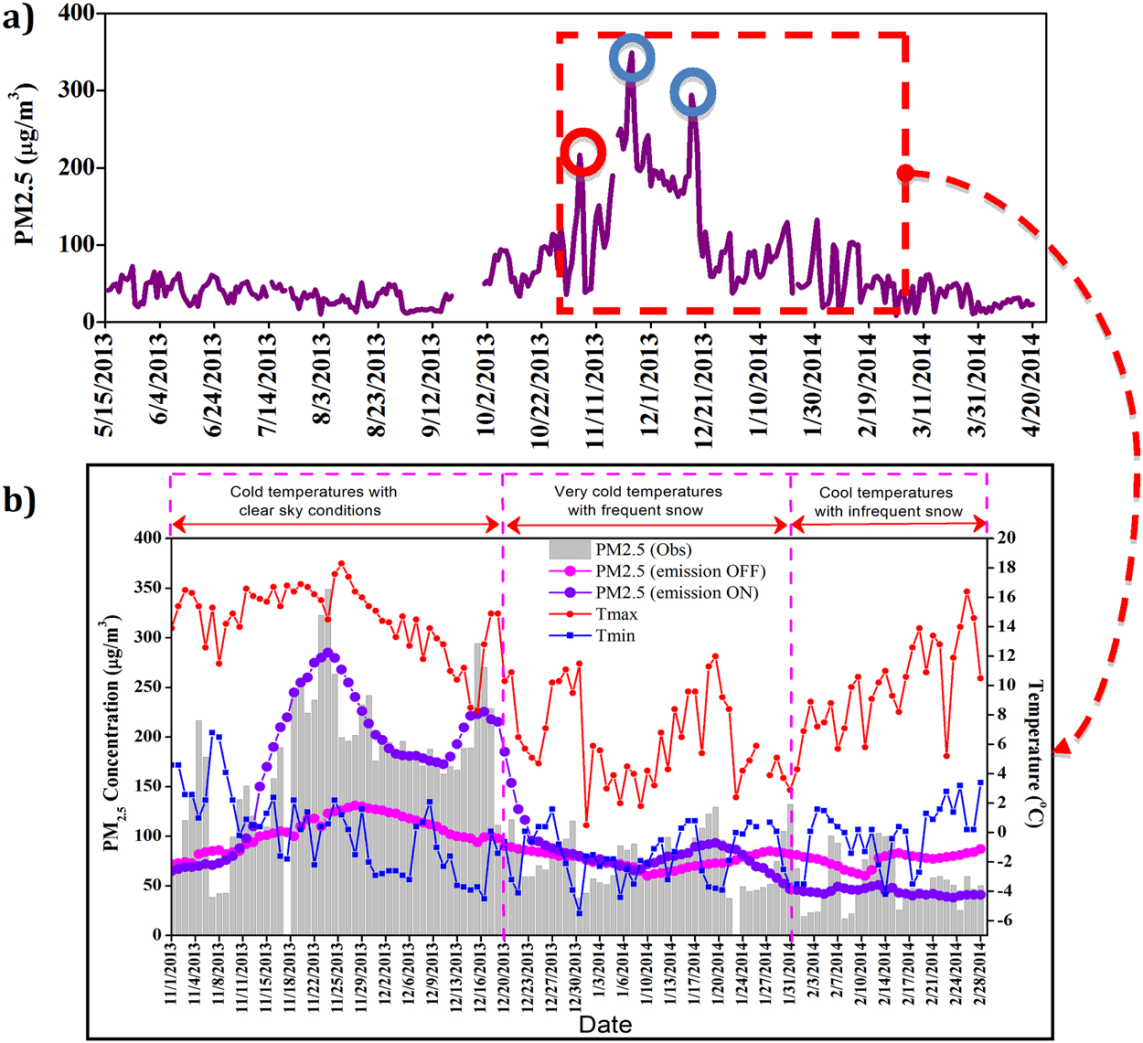
(**a**) 24 hour average PM_2.5_ concentration measured at a point in Kashmir Valley for the period May 2013-April 2014. Blue circles indicate coldest days. Red circle indicates Diwali festival day. (**b**) Offset figure of (**a**), winter variability analysis with model simulations and maximum and minimum temperatures. Observed and model simulated values of PM_2.5_ along with maximum and minimum temperatures are shown for the winter period. The vertical grey bars in (**b**) represent observed 24-hour average levels. The time series of model simulated PM_2.5_ with local emissions ON and OFF are also shown in this figure.

Time series analysis of PM_2.5_ daily average from 15 May 2013 to 20 April 2014 was performed (Fig. 3a). The time period was sub-grouped into four different seasons of the Kashmir Valley, namely, Summer (MAM), Autumn (SO), Spring (JJA) and Winter (NDJF). During the summer, autumn and spring seasons the values of PM_2.5_ are between the range 20–50 µg/m^3^, which are well within the Indian permissible limits of 60 µg/m^3^ (Revised National Ambient Air Quality Standards (MoEF notification G.S.R 826 (E), dated 16.11.2009)). Although the spring and autumn (September-October) seasons are fairly cold, people make use of the conventional energy sources only for warming water. In summer (June-July-August) the discharge from rivers is high, leading to a peak in the generation of hydel power and decline in the use of conventional energy sources. The emissions from coal and fuel wood burning are low (Fig. 2b), bringing down the levels of PM_2.5_ well within the permissible limits during spring, autumn and summer. Unlike summer months, the PM_2.5_ levels exceed the permissible limits on a consistent basis during winter months. Hence, in the rest of this paper; we focus only over the winter period. The value of PM_2.5_ starts to rise in the month of October and remains elevated above 60 µg/m^3^ until February. We also observe significant variability in the PM_2.5_ levels amongst winter months. In order to understand the various phenomena responsible for the elevated levels and variability in the PM_2.5_ concentrations, we have made WRF-Chem model simulations for the winter period using the emission inventory developed in this work and made sensitivity simulations.

**Figure 4:**
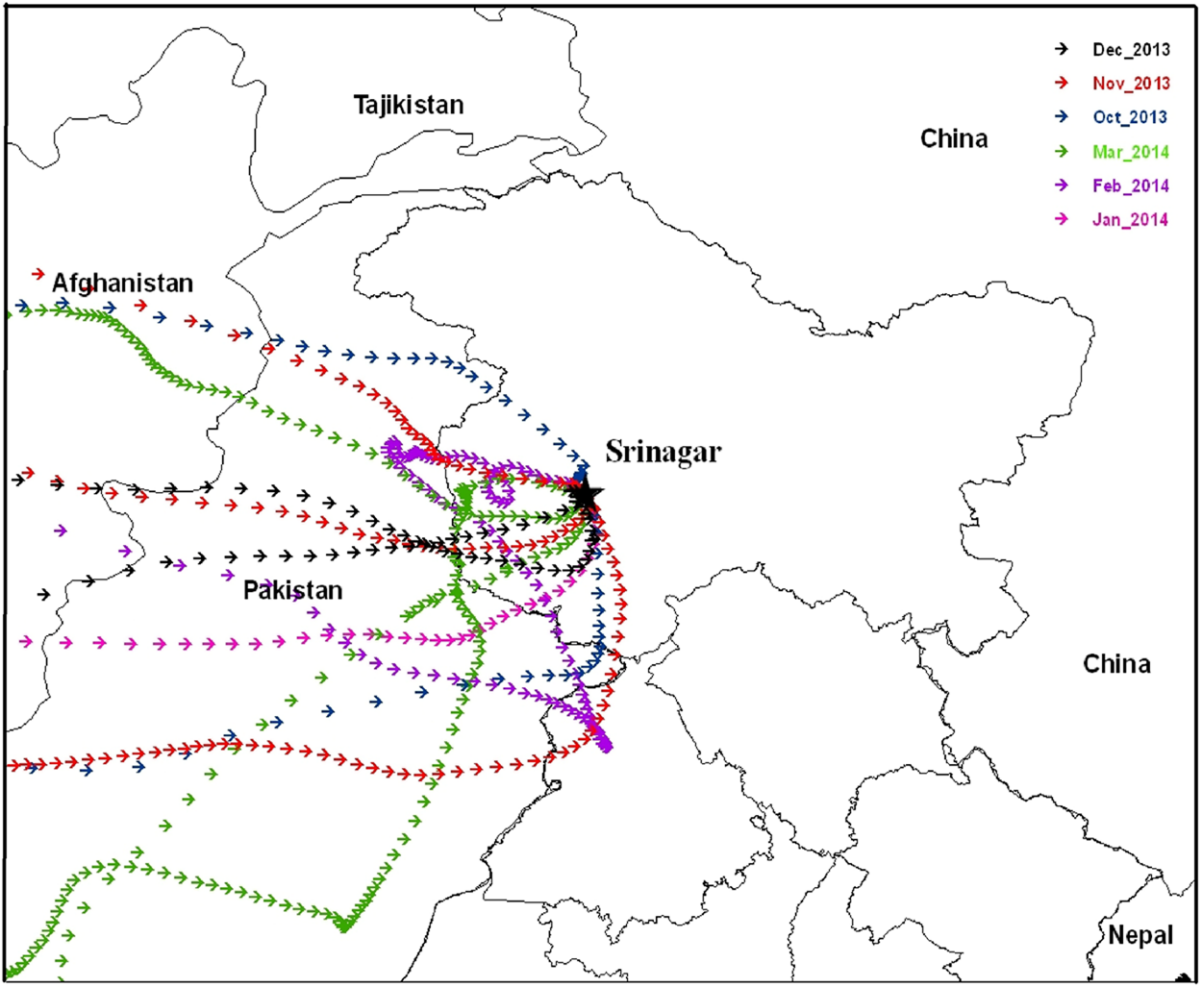
Map showing back trajectory analysis over Kashmir Valley at Srinagar. The backward trajectories were created using HYbrid Single-Particle Lagrangian Integrated Trajectory (HYSPLIT) model version 4 of NOAA’s-ARL (National Oceanic and Atmospheric Administration’s Air Resources Laboratory) [*Draxler and Hess 1998; Draxler and Rolph 2003; Rolph 2003*.]. The back trajectories were superimposed on geographical map prepared using ArcGIS version 9.3.1 software by Environmental Systems Research Institute (ESRI, www.esri.com) and the figure is used herein under license.

Based on the local weather conditions and wind direction pattern, we have divided the entire winter season into three regimes (w1, w2, w3), as shown by vertical dotted pink lines in Fig. 3b, in order to explain the variability in the levels of PM_2.5_. Emissions from anthropogenic activities are very high in winter. Most of the power supply in the valley comes from hydel power generation. During winter the rivers freeze and there is a sharp decline in the flow of water, making power supply minimum when required the most. People then make use of conventional energy sources as an alternative to state run power supply. They use traditional firepots known as kangris, ignited by charcoal, to keep themselves warm outside and burn firewood in a hollow room known as hamam for central indoor heating. The usage of biofuels and enhanced emission peaks in the winter and mostly remains the same as shown in Fig. 3b with little variability amongst the winter months. Back trajectory analysis for different winter periods indicates that majority of the time the air mass originating from Indo-Gangetic Plain (IGP) region and that from the borders of Afghanistan and Pakistan is flowing towards study area which is not the case for other seasons (Fig. 4). Hence, in general there is a larger contribution from the medium-range transport of pollution from distance sources during winter season almost equally in 3 regimes.

To further investigate the matter, sensitivity runs are performed by switching on and off the local emissions to understand the role of transport in different seasons, which confirms the observed findings. The normal run (Scenario A) is able to reproduce the observed features (Fig. 3b) whereas Scenario C is unable to capture the same. It is clear from Fig. 3b that although local emissions and medium-range transport contributes significantly during winter months in the elevated level of PM_2.5_, it is local weather which is mainly responsible in the huge variability observed within the winter months among these 3 regimes as indicated in Fig. 3b. The first regime (w1), from November 1 to December 20, experiences cool temperatures and clear sky conditions. During this period, elevated levels of PM_2.5_ have been observed. The temperatures being low, the height of boundary layer falls to lower layers and it traps the pollutants close to the earth’s surface. We observed that during this regime the winds are westerly, which dump loads of dust from the dry regions of Afghanistan and surrounding areas in the Valley (Fig. 4). These conditions are very favorable for the formation of secondary organic aerosols (SOA)^19^, thus increasing the levels of PM_2.5_ in the range 70–348 μg/m³.

We observed peaks on 24 November and 16 December due to very low temperatures. The values of PM_2.5_ rise as high as 348 μg/m³ and 294 μg/m³ on respective days. There is also a sudden rise in the mass concentration of PM_2.5_ on 5 November. This peak is due to the emissions from the pyrotechnic displays during Diwali. Since it is a two-day lag peak, the emissions are attributed to come from local as well as the neighboring areas. During the second regime (w2), from 21 December to 31 January, the valley experiences harsh winter characterized by frequent snowfall and very cold temperatures, locally known as Chillai Kalan. Due to extremely cold temperatures the boundary layer further lowers, increasing the concentration of pollutants near the surface. But the snowfall frequently washes out local emissions and dust particles transported by intercontinental winds, thus suppressing the levels of PM_2.5_ in the range 40–130 μg/m³ during the period of Chillai Kalan. In the month of February, the third regime (w3), most of the wind comes from the central part of India which is cleaner compared to the winds coming from the dry regions to the west in other winter months. These clean winds and the rise in temperature maintain low levels of PM_2.5_ between 30–100 μg/m³, which is still higher than the prescribed limits i.e. 60 μg/m³.

## Conclusions

The long-term observation of fine particulate matter (PM_2.5_) at Srinagar (Kashmir Valley-relatively cleaner environment) in India was analyzed with model simulations during the study period 2013–14. Results indicate that the air quality of Srinagar city deteriorates significantly in particular during winter period, where level of PM_2.5_ touches a peak value of 348 μg/m³ against the Indian permissible limit of 60 μg/m³. The emissions due to domestic coal usage are found to be 1246.4 tons/year that accounts for 84% of the total annual emissions. The chemistry transport model (WRF-Chem) reproduces the seasonal variability reasonably well. We can therefore conclude that although the fundamental reason for the high pollution during winter season is the medium range transport of air mass from the pollution rich region and elevated level of emissions from coal and bio-fuel, the huge variability within the four winter months can only be explained by local weather which plays a decisive role in deciding the air quality of the region. The cold temperature with dry weather keeps the boundary layer near the surface during nights and hence responsible for highest pollution in regime period w1. Although the local biofuel emissions increases during regime w2 and temperature goes further colder but frequent snowfall washes away and keeps in check the expected high level of PM_2.5_. When the winter period comes to an end in regime w3, temperature starts to go up and usage of biofuel decreases. During this period winds start to blow from cleaner areas and hence level of PM_2.5_ starts to decline and sets around 50 μg/m³.

### Data availability statement

The datasets generated during and/or analysed during the current study are available from the corresponding author on reasonable request.
